# Ethnic- and sex-specific associations between plasma fatty acids and markers of insulin resistance in healthy young adults

**DOI:** 10.1186/1743-7075-10-42

**Published:** 2013-06-17

**Authors:** Jessica C Ralston, Michael A Zulyniak, Daiva E Nielsen, Shannon Clarke, Alaa Badawi, Ahmed El-Sohemy, David WL Ma, David M Mutch

**Affiliations:** 1 Department of Human Health and Nutritional Sciences, University of Guelph, Guelph, Ontario N1G 2W1, Canada; 2 Department of Nutritional Sciences, University of Toronto, 150 College Street, Toronto, Ontario M5S 3E2, Canada; 3 Public Health Agency of Canada, Office of Biotechnology, Genomics and Population Health, Toronto M5V 3L7, Canada

**Keywords:** Caucasian, South Asian, East Asian, HOMA-IR, Fasting glucose and fasting insulin

## Abstract

**Background:**

Although evidence indicates that fatty acids (FA) can affect insulin resistance (IR), not all FA contribute equally to the process. Indeed, monounsaturated FA (MUFA) and polyunsaturated FA (PUFA) are reported to reduce IR, whereas saturated FA (SFA) and trans FA appear to increase IR. However, it is not yet clear how individual FA are associated with markers of IR, and whether these relationships are influenced by ethnicity and/or sex. Therefore, the goal of this study was to examine the ethnic- and sex-specific relationships between plasma FA and markers of IR in a cohort of healthy young Caucasian, East Asian, and South Asian adults.

**Methods:**

Gas chromatography was used to quantify fasting plasma FA from young Canadian adults (22.6 ± 0.1 yrs) of Caucasian (n = 461), East Asian (n = 362), or South Asian (n = 104) descent. Linear regression models were used to investigate associations between plasma FA and markers of IR (i.e. fasting insulin, glucose, and HOMA-IR) according to ethnicity and sex.

**Results:**

Numerous significant associations (*P* < 0.05, adjusted for multiple testing) were identified between individual FA and markers of IR, with the majority identified in Caucasians. For SFA, positive associations were found between 14:0 and fasting insulin and HOMA-IR in Caucasian and East Asian populations, and 18:0 and fasting glucose in Caucasians only. Several positive associations were also found for specific MUFA (18:1t11 and 18:1t6-8 with HOMA-IR, and 18:1c9 with fasting glucose) and PUFA (18:2n6 with fasting glucose and 18:2c9t11 with HOMA-IR) in Caucasian adults only. Most of the aforementioned associations were stronger in males compared to females. Interestingly, no significant associations were found between FA and markers of IR in South Asian adults.

**Conclusions:**

We report numerous associations between plasma FA and markers of IR in Caucasian and East Asian populations, but not in South Asian individuals. Furthermore, these associations appeared to be more robust in men. This demonstrates the importance of investigating associations between FA and markers of IR in an ethnic- and sex-specific manner in order to better understand the contribution of plasma FA to the development of IR and type-2 diabetes.

## Background

Type-2 diabetes (T2D) is a health concern affecting 285 million individuals worldwide, and is expected to affect 439 million individuals by 2030 [1]. T2D is a chronic metabolic disorder characterized by insufficient insulin production, which is preceded by a state of insulin resistance (IR) [2,3]. It is well established that high fat diets contribute to the development of IR [4,5]; however, not all fatty acids (FA) influence IR equally. For example, high circulating levels of saturated fatty acids (SFA) and n-6 polyunsaturated fatty acids (PUFA) are associated with elevated fasting levels of insulin and glucose [6-8]. Interestingly, these same FA were reported to be more abundant in diabetic patients compared to healthy individuals [8]. Conversely, certain n-3 PUFA such as α-linolenic acid (ALA), eicosapentaenoic acid (EPA) and docosahexaenoic acid (DHA) have been shown to improve glucose homeostasis and provide protective effects against the development of IR by increasing production of insulin-sensitizing adipokines (e.g. adiponectin) and reducing the pro-inflammatory state of adipocytes and macrophages [9-11]. This reinforces that the associations between individual FA and the development of IR are distinct.

While several excellent cross-sectional studies have examined the relationships between FA and markers of cardiometabolic risk [12-15], it is notable that most of these previous studies have not investigated to what extent these associations vary with ethnicity. This is significant as it is now acknowledged that specific ethnicities may be more susceptible to develop metabolic diseases than others [16]. For example, associations between common inflammatory markers (e.g. C-reactive protein and interleukin-6) and IR appear to vary according to ethnicity [16], which could contribute to ethnic-specific health disparities. Additionally, individuals of African, Latino, Japanese, and Hawaiian descent residing in America are more susceptible to developing diabetes than Americans of European descent [17]. Recent work by Goff *et al*. also demonstrated that individuals of South Asian, African-Caribbean and European origins differ significantly in their habitual dietary intake (e.g. total daily energy intake, % fat intake, and % sugar intake) and that these characteristics are associated with differences in basal insulin sensitivity and secretion [18]. Such ethnic-specific differences are likely not explained by a single factor, but rather a myriad of complex gene-environment interactions. As such, there is a need to conduct further analyses in order to elucidate whether the relationships between FA and markers of IR differ between ethnicities.

Furthermore, evidence also demonstrates that plasma FA profiles vary between men and women [19,20]. These dissimilarities in plasma FA are often attributed to fundamental differences between men and women, such as total adiposity and lean muscle mass, location of fat deposition (i.e. visceral versus subcutaneous), and systemic hormone concentrations [19,21]. This suggests that greater insight regarding the relationships between FA and markers of IR may be obtained by further stratifying each ethnicity by sex [22]. Therefore, the goal of the present study was to conduct a cross-sectional analysis in young Canadian adults of different ethnicity in order to uncover ethnic- and sex-specific relationships between fasting plasma FA concentrations and markers of IR.

## Methods

### Study participants

Participants recruited from the University of Toronto campus (2004–2009) for the Toronto Nutrigenomics and Health (TNH) study were used for the current investigation [23,24]. Participants were between the ages of 20–29 yrs and classified as either Caucasian (n = 461; 132 men / 329 women), East Asian (n = 362; 95 men / 267 women), or South Asian (n = 104; 41 men / 63 women) by self-reported ethnicity. Participants were excluded from the investigation if they had a body mass index (BMI) greater than 30 kg/m^2^, did not complete a food frequency questionnaire (FFQ), or were diagnosed with diabetes or cancer. The TNH study was approved by the Research Ethics Boards at the University of Toronto and the University of Guelph. All subjects provided informed written consent.

### Clinical measurements

Plasma samples were collected following a 12 hour overnight fast, and were used for analysis of glucose and insulin at LifeLabs Laboratories (Toronto, Canada). The homeostatic model assessment of insulin resistance (HOMA-IR) was calculated using the HOMA Calculator v2.2.2 (http://www.dtu.ox.ac.uk/homacalculator/index.php).

### Food frequency and health questionnaires

Subjects were asked to complete a one-month 196-item food frequency questionnaire (FFQ), adapted from the Willet questionnaire [23,25]. This FFQ data was used to estimate total dietary intake of fat (in grams) per day. Subjects also completed lifestyle and general health questionnaires, which were used to calculate a physical activity (PA) score.

### Plasma fatty acid analysis

Fasting plasma samples were assessed using gas chromatography (GC) as previously described [26]. Briefly, 5 μg of a C17:0 internal standard was added to plasma samples and mixed with a 2:1 chloroform:methanol solution containing KCl. Following an overnight incubation at 4°C, samples were centrifuged and the organic layer was dried under a gentle stream of nitrogen gas. Extracted lipids were saponified with 0.5 M KOH in methanol for 1 hour at 100°C, and cooled for 10 minutes at room temperature. 14% BF_3_-MeOH and hexane were added to samples, prior to methylation, for 1 hour at 100°C. After samples had cooled, double-distilled water was added, and samples were centrifuged at 1000 rpm for 10 minutes. The upper-layer was extracted and dried under nitrogen gas, prior to reconstitution in hexane. Samples were analyzed using an Agilent Technologies 7890A GC system (Agilent Technologies, Mississauga, CA). All FA peaks were identified by comparison to retention times of FA methyl ester standards. Only those FA that were consistently detected across all plasma samples were considered for the current study (i.e. 24 of the 62 FA that can be measured by GC). Absolute FA values were calculated by comparing individual FA peaks to the internal standard and are reported as μg FA / mL plasma ± SE.

### Statistical analysis

All statistical analyses were conducted with JMP Genomics software Version 5.1 (SAS Institute, Cary, NC). The Shapiro-Wilk test was used to assess the distribution of all variables. Fasting insulin was subsequently log transformed for all analyses. Fourteen outliers were identified by performing a Jackknife test and removed prior to the analyses. Baseline anthropometric, clinical, and FA data were examined using a Kruskal-Wallis analysis of variation (ANOVA) test followed by a post-hoc Mann–Whitney-Wilcoxon test. Individual associations between FA and markers of IR (i.e. fasting insulin and glucose, and HOMA-IR) were examined using mixed-effects linear regression models, which accounted for both fixed effects (age, sex, BMI, PA, and average daily fat intake (in grams) per day) and one random effect (date of GC processing). For analyses of sex-specific associations, sex was removed from the aforementioned list of variables. We used a two-step process to determine statistical significance: (1) ethnic-specific associations between FA and markers of IR were considered significant only if they satisfied a Bonferroni correction for multiple testing; followed by (2) an investigation of sex-specific differences for those FA that were statistically significant in the previous step. A *P*-value < 0.05 was considered statistically significant.

## Results

### Study participant characteristics

Anthropometric and clinical characteristics of the 927 study participants are shown in Table 1. Baseline FA levels for the three ethnicities are provided in Table 2. The major plasma FA in all three ethnicities were 16:0, 18:1n9, and 18:2n6, as expected. All subjects were considered to be within the normal healthy range for all parameters. No subjects were diabetic or affected by cancer, and only 2 subjects were smokers.

**Table 1:** Study population characteristics

	**Caucasian**	**East Asian**	**South Asian**
Number (M/F)	461 (132/329)	362 (95/267)	104 (41/63)
Age (yrs)	23.1 ± 0.1^a^	22.1 ± 0.1^b^	22.3 ± 0.2^b^
BMI (kg/m^2^)	22.7 ± 0.1^a^	21.6 ± 0.1^b^	22.8 ± 0.3^a^
Total fat (g/d)	68.4 ± 1.2^a^	61.6 ± 1.3^b^	60.7 ± 2.7^b^
Log insulin (pmol/L)	1.57 ± 0.01^a^	1.59 ± 0.01^a^	1.71 ± 0.02^b^
Glucose (mmol/L)	4.72 ± 0.02^a^	4.78 ± 0.02^a^	4.90 ± 0.03^b^
HOMA-IR	0.78 ± 0.02^a^	0.82 ± 0.02^a^	1.08 ± 0.05^b^

A Kruskal-Wallis analysis of variation (ANOVA) test followed by a post-hoc Mann–Whitney-Wilcoxon test was used to examine each parameter in the three ethnicities. Ethnic groups with different superscript letters (e.g. a or b) are statistically different (*P* < 0.05) for a given parameter. All data is represented as mean ± standard error. M/F = male/female; BMI = body mass index; HOMA-IR = Homeostasis Model of Assessment of Insulin Resistance.

**Table 2:** **Baseline fatty acid levels in healthy young adults of Caucasian**, **East Asian**, **and South Asian descent**

	**Mean concentration** (**μg**/**mL**)		
	**Caucasian**	**East Asian**	**South Asian**
	(**n** = **461**)	(**n** = **362**)	(**n** = **104**)
*Saturated fatty acids*			
14:0	16.31 ± 0.48^a^	13.67 ± 0.54^b^	15.26 ± 1.00^a,b^
15:0	4.59 ± 0.09^a^	3.85 ± 0.11^b^	4.21 ± 0.20^a^
16:0	422.74 ± 6.38^a,b^	420.03 ± 7.20^a^	400.01 ± 13.43^b^
18:0	127.38 ± 1.61^a^	135.49 ± 1.82^b^	130.87 ± 3.39^a,b^
*Monounsaturated fatty acids*			
16:1c9	37.86 ± 0.96^a^	34.71 ± 1.08^a^	29.91 ± 2.02^b^
18:1t6-8	2.08 ± 0.08^a^	1.96 ± 0.08^a^	2.03 ± 0.16^a^
18:1t9	4.34 ± 0.13^a^	4.06 ± 0.15^a^	4.24 ± 0.28^a^
18:1t10	4.37 ± 0.14^a^	4.12 ± 0.16^a^	4.08 ± 0.30^a^
18:1t11	4.52 ± 0.12^a^	4.06 ± 0.13^b^	4.40 ± 0.24^a^
18:1c9	365.19 ± 7.55^a^	376.02 ± 8.52^a^	341.75 ± 15.89^b^
18:1c11	32.68 ± 1.03^a^	35.55 ± 1.16^b^	29.22 ± 2.17^c^
18:1c12	4.50 ± 0.13^a^	4.28 ± 0.15^a^	3.76 ± 0.28^a^
*Polyunsaturated fatty acids*			
18:2c9t12	4.62 ± 0.18^a^	4.04 ± 0.20^b^	4.10 ± 0.37^a,b^
18:2t9c12	2.58 ± 0.05^a^	2.62 ± 0.06^a^	2.57 ± 0.11^a^
18:2n6	592.14 ± 7.58^a^	645.99 ± 8.56^b^	606.51 ± 15.97^a^
18:3n6	6.55 ± 0.18^a^	5.38 ± 0.20^b^	7.94 ± 0.38^c^
18:3n3	13.52 ± 0.30^a^	14.99 ± 0.34^b^	15.54 ± 0.64^b^
18:2c9t11	4.70 ± 0.10^a^	3.86 ± 0.11^b^	4.28 ± 0.20^b^
20:2n6	4.09 ± 0.08^a^	4.26 ± 0.09^b^	3.68 ± 0.17^c^
20:3n6	26.01 ± 0.49^a^	20.19 ± 0.56^b^	24.06 ± 1.04^a^
20:4n6	117.11 ± 1.72^a^	111.56 ± 1.94^b^	125.62 ± 3.61^a^
20:5n3	10.77 ± 0.39^a^	12.63 ± 0.44^b^	10.89 ± 0.82^a,b^
22:5n3	6.72 ± 0.14^a^	7.28 ± 0.16^b^	7.23 ± 0.30^a,b^
22:6n3	27.54 ± 0.57^a^	33.86 ± 0.64^b^	25.51 ± 1.20^a^

A Kruskal-Wallis analysis of variation (ANOVA) test followed by a post-hoc Mann–Whitney-Wilcoxon test was used to examine each fatty acid in the three ethnicities. Ethnic groups with different superscript letters (e.g. a or b) are statistically different (*P* < 0.05) for a given parameter. All data is represented as mean μg/mL ± standard error.

### Associations between fatty acids and markers of insulin resistance

Our GC platform is able to detect 62 distinct FA; however, for the current study we only considered FA that were consistently detected in all plasma samples. Using this judicious approach, we investigated associations between 24 individual FA (listed in Table 2) and various markers of IR in three ethnic groups of healthy young adults. Interestingly, all of the statistically significant associations identified indicated positive relationships between FA and markers of IR. These significant associations were further investigated in a sex-specific manner.

#### Saturated fatty acids (SFA)

Several SFA were examined (14:0, 15:0, 16:0, 18:0) for associations with markers of IR (Tables 3, 4 and 5). In the Caucasian population, plasma 14:0 levels were positively associated with both fasting insulin (r^2^ = 0.19; *P* = 0.0001) and HOMA-IR (r^2^ = 0.17; *P* < 0.0001). Similar associations were also seen in the East Asian population, where 14:0 was positively correlated with both fasting insulin (r^2^ = 0.13; *P* = 0.0088) and HOMA-IR (r^2^ = 0.11; *P* = 0.0024); however, these associations were borderline significant when adjusting for multiple testing. After separating the Caucasian and East Asian populations by sex, 14:0 remained positively associated with insulin in Caucasian men and women (r^2^ = 0.31; *P* = 0.0049, and r^2^ = 0.13; *P* = 0.0049, respectively) and East Asian men (r^2^ = 0.15; *P* = 0.0233). The relationship between 14:0 and insulin was borderline significant in East Asian females (r^2^ = 0.14; *P* = 0.0515). For HOMA-IR, 14:0 remained positively associated in both Caucasian men and women (r^2^ = 0.33; *P* = 0.0006, and r^2^ = 0.12; *P* = 0.0031, respectively) and East Asian men and women (r^2^ = 0.17; *P* = 0.0259, and r^2^ = 0.10; *P* = 0.0142, respectively). No associations were seen with 14:0 in the South Asian group.

**Table 3:** **Associations between fatty acids and markers of insulin resistance in healthy young Caucasian adults** (**n** = **461**)

	**HOMA**-**IR**		**Insulin** (**log**)		**Glucose**	
	**r**^**2**^	**P**-**value**	**r**^**2**^	**P**-**value**	**r**^**2**^	**P**-**value**
14:0	0.17	<**0**.**0001**	0.19	**0**.**0001**	0.08	0.0390
15:0	0.16	0.0022	0.17	0.0073	0.08	0.0376
16:0	0.15	0.0089	0.17	0.0780	0.08	0.0214
16:1c9	0.15	0.1443	0.16	0.5274	0.07	0.3644
18:0	0.15	0.0216	0.16	0.1018	0.10	**0**.**0007**
18:1t6-8	0.16	**0**.**0015**	0.17	0.0437	0.07	0.4216
18:1t9	0.15	0.0334	0.16	0.1003	0.07	0.3634
18:1t10	0.16	0.0041	0.17	0.0089	0.08	0.1178
18:1t11	0.16	**0**.**0006**	0.18	0.0029	0.09	0.0136
18:1c9	0.15	0.0122	0.16	0.0848	0.09	**0**.**0017**
18:1c11	0.14	0.2349	0.16	0.6690	0.08	0.0314
18:1c12	0.16	0.0038	0.17	0.0118	0.07	0.4610
18:2c9t12	0.15	0.0405	0.17	0.0690	0.07	0.3508
18:2t9c12	0.15	0.0394	0.16	0.1262	0.15	0.0754
18:2n6	0.15	0.1586	0.16	0.4673	0.10	**0**.**0005**
18:3n6	0.14	0.3024	0.16	0.5425	0.08	0.0865
18:3n3	0.15	0.0239	0.16	0.0804	0.09	0.0051
18:2c9t11	0.16	**0**.**0020**	0.17	0.0073	0.08	0.0480
20:2n6	0.15	0.0731	0.16	0.2296	0.08	0.0915
20:3n6	0.15	0.0135	0.17	0.0609	0.07	0.3305
20:4n6	0.14	0.7651	0.16	0.7115	0.08	0.0125
20:5n3	0.15	0.1953	0.16	0.4116	0.07	0.2991
22:5n3	0.14	0.5481	0.16	0.722	0.07	0.2311
22:6n3	0.14	0.6422	0.16	0.3711	0.07	0.992

Bold font indicates fatty acids which demonstrated statistically significant associations that satisfied a Bonferroni correction for multiple testing. r^2^ = Pearson’s correlation coefficient. HOMA-IR = Homeostasis Model of Assessment of Insulin Resistance.

**Table 4:** **Associations between fatty acids and markers of insulin resistance in healthy young East Asian adults** (**n** = **362**)

	**HOMA**-**IR**		**Insulin** (**log**)		**Glucose**	
	**r**^**2**^	**P**-**value**	**r**^**2**^	**P**-**value**	**r**^**2**^	**P**-**value**
14:0	0.11	0.0024	0.13	0.0088	0.10	0.0614
15:0	0.09	0.1060	0.12	0.1775	0.10	0.1762
16:0	0.09	0.1127	0.12	0.2928	0.10	0.2815
16:1c9	0.21	0.0873	0.12	0.2311	0.10	0.0785
18:0	0.09	0.1854	0.12	0.5403	0.10	0.2818
18:1t6-8	0.08	0.4737	0.11	0.6167	0.11	0.0565
18:1t9	0.09	0.2372	0.12	0.4811	0.11	0.0867
18:1t10	0.09	0.2883	0.12	0.4064	0.10	0.1989
18:1t11	0.08	0.8989	0.11	0.9035	0.10	0.2031
18:1c9	0.09	0.2495	0.12	0.2969	0.11	0.0355
18:1c11	0.09	0.3086	0.12	0.1099	0.10	0.4933
18:1c12	0.08	0.3911	0.12	0.3966	0.11	0.0535
18:2c9t12	0.09	0.0666	0.12	0.1023	0.10	0.2928
18:2t9c12	0.09	0.1503	0.12	0.2362	0.10	0.6072
18:2n6	0.08	0.5441	0.12	0.3801	0.10	0.8027
18:3n6	0.09	0.2841	0.12	0.2322	0.11	0.0171
18:3n3	0.09	0.1225	0.12	0.1447	0.10	0.5359
18:2c9t11	0.09	0.2516	0.12	0.3460	0.12	0.0085
20:2n6	0.08	0.4447	0.12	0.3905	0.10	0.8557
20:3n6	0.09	0.0508	0.13	0.0354	0.10	0.0894
20:4n6	0.08	0.7148	0.11	0.9917	0.10	0.3656
20:5n3	0.08	0.4941	0.12	0.4479	0.10	0.8266
22:5n3	0.08	0.9893	0.11	0.9204	0.10	0.6846
22:6n3	0.08	0.6374	0.12	0.5313	0.10	0.4428

r^2^ = Pearson’s correlation coefficient. HOMA-IR = Homeostasis Model of Assessment of Insulin Resistance.

**Table 5:** **Associations between fatty acids and markers of insulin resistance in healthy young South Asian adults** (**n** = **104**)

	**HOMA**-**IR**		**Insulin** (**log**)		**Glucose**	
	**r**^**2**^	**P**-**value**	**r**^**2**^	**P**-**value**	**r**^**2**^	**P**-**value**
14:0	0.15	0.0518	0.10	0.0897	0.13	0.0164
15:0	0.12	0.4915	0.08	0.6001	0.08	0.5987
16:0	0.12	0.4176	0.08	0.6002	0.09	0.2739
16:1c9	0.12	0.6737	0.08	0.7724	0.09	0.2273
18:0	0.12	0.8328	0.08	0.9534	0.09	0.3685
18:1t6-8	0.12	0.4670	0.08	0.4406	0.14	0.0109
18:1t9	0.12	0.3505	0.08	0.2504	0.13	0.0145
18:1t10	0.11	0.5304	0.07	0.5077	0.12	0.2557
18:1t11	0.13	0.4832	0.08	0.5813	0.09	0.3856
18:1c9	0.13	0.2700	0.09	0.3395	0.08	0.3988
18:1c11	0.12	0.3661	0.08	0.4598	0.08	0.7893
18:1c12	0.12	0.8614	0.08	0.9561	0.08	0.7816
18:2c9t12	0.12	0.4865	0.08	0.7288	0.09	0.3157
18:2t9c12	0.12	0.6536	0.08	0.5053	0.09	0.2049
18:2n6	0.12	0.5885	0.08	0.7771	0.08	0.6857
18:3n6	0.12	0.5773	0.08	0.8202	0.09	0.2618
18:3n3	0.19	0.0057	0.14	0.0093	0.09	0.2911
18:2c9t11	0.12	0.2849	0.08	0.3788	0.04	0.3001
20:2n6	0.13	0.2273	0.09	0.2951	0.03	0.9353
20:3n6	0.13	0.1943	0.09	0.2922	0.03	0.9261
20:4n6	0.13	0.2706	0.10	0.1549	0.03	0.7612
20:5n3	0.12	0.8226	0.08	0.8758	0.04	0.5325
22:5n3	0.12	0.6271	0.08	0.7156	0.04	0.3235
22:6n3	0.12	0.3889	0.08	0.3763	0.04	0.5587

r^2^ = Pearson’s correlation coefficient. HOMA-IR = Homeostasis Model of Assessment of Insulin Resistance.

We also found that 18:0 was positively associated with fasting glucose levels (r^2^ = 0.10; *P* = 0.0007) in the Caucasian population. Furthermore, this association remained significant after separating Caucasians into men (r^2^ = 0.15; *P* = 0.0112) and women (r^2^ = 0.05; *P* = 0.0225). No significant associations were found between 18:0 and markers of IR in the East Asian or South Asian populations.

No associations were identified between plasma 15:0 or 16:0 with markers of IR in the three ethnicities examined.

#### Monounsaturated fatty acids (MUFA)

Numerous MUFA were examined for associations with markers of IR, including 16:1c9, 18:1t6-8, 18:1t9, 18:1t10, 18:1t11, 18:1c9, 18:1c11 and 18:1c12 (Tables 3, 4 and 5). Of the aforementioned MUFA, 18:1t11 was positively associated with HOMA-IR in the Caucasian population (r^2^ = 0.16; *P* = 0.0006). Separating Caucasians by sex revealed that the positive association between 18:1t11 and HOMA-IR remained significant in both men (r^2^ = 0.31; *P* = 0.0049) and women (r^2^ = 0.12; *P* = 0.0125). No significant associations were detected between 18:1t11 and markers of IR in East Asians or South Asians. Additionally in the Caucasian population, 18:1t6-8 was positively associated with HOMA-IR (r^2^ = 0.16; *P* = 0.0015), and this relationship also remained significant in both Caucasian men (r^2^ = 0.30; *P* = 0.0167) and women (r^2^ = 0.12; *P* = 0.0317). Similarly, this relationship was not observed in East Asians or South Asians.

Next, we found that 18:1c9 was positively associated with fasting glucose in the Caucasian population (r^2^ = 0.09; *P* = 0.0017); however, this relationship remained significant only in men (r^2^ = 0.20; *P* = 0.0004), and not women (r^2^ = 0.04; *P* = 0.1294), when separated by sex. No associations were found between 18:1c9 and markers of IR in East Asians or South Asians.

No significant associations were detected between markers of IR and 16:1c9, 18:1t9, 18:1t10, 18:1c11 or 18:1c12.

#### Polyunsaturated fatty acids (PUFA)

Twelve PUFA were examined for their associations with markers of IR (18:2c9t12, 18:2t9c12, 18:2n6, 18:3n6, 18:3n3, 18:2c9t11, 20:2n6, 20:3n6, 20:4n6, 20:5n3, 22:5n3, 22:6n3) (Tables 3, 4 and 5). Significant associations with PUFA were only detected in the Caucasian population. 18:2n6 was positively associated with fasting glucose (r^2^ = 0.10; *P* = 0.0005), while 18:2c9t11 was positively associated with HOMA-IR (r^2^ = 0.16; *P* = 0.0020). Further sex-specific investigations demonstrated that the positive association between fasting glucose and 18:2n6 remained significant in both men (r^2^ = 0.15; *P* = 0.0163) and women (r^2^ = 0.05; *P* = 0.0086). The positive associations between 18:2c9t11 and HOMA-IR also remained significant in both men (r^2^ = 0.30; *P* = 0.0097) and women (r^2^ = 0.11; *P* = 0.0281). No associations with these PUFA were found in South Asians or East Asians.

No significant associations with markers of IR were found for 18:2c9t12, 18:2t9c12, 18:3n6, 18:3n3, 20:2n6, 20:3n6, 20:4n6, 20:5n3, 22:5n3, or 22:6n3.

## Discussion

In this study, we conducted a cross-sectional analysis to determine whether the relationship between individual FA and markers of IR varied in an ethnic- and/or sex-specific manner in a population of healthy young Canadian adults. Previous investigations have examined the relationships between FA and markers of IR in older adults, as well as in unhealthy men and women from geographically distinct populations [18,27]; however, we believe that this is the first study to investigate these relationships in a population of healthy young men and women differing in ethnicity but living in the same geographical region. This enabled us to observe ethnic-specific associations between FA and markers of IR while avoiding the recognized confounder of geographical location. Indeed, we observed that numerous associations between FA and markers of IR were only seen in certain ethnicities. We hypothesize that this could be due to underlying genetic differences that could influence FA composition in an ethnic-specific manner [28,29]. Moreover, we also examined whether sex could influence the relationships between FA and markers of IR within each ethnicity. We noted that although men and women showed similar relationships between FA and markers of IR, the correlations in men were typically stronger compared to women. This aligns with previous work by Frias *et al*., who demonstrated that elevations of plasma free FA concentrations impaired insulin sensitivity in the tissues of men, but not women [19]. This suggests that underlying sex-specific characteristics (e.g. hormone levels, adiposity, lifestyle habits, and quantity of muscle mass) are influencing this relationship [30,31]. In short, our study has identified numerous ethnic-specific associations between FA and markers of IR, and the majority of these associations appeared to be more robust in men.

Of additional interest, we noted that South Asian subjects had significantly higher levels of fasting insulin and glucose concentrations compared to Caucasian subjects despite having a similar BMI (22.8 ± 0.3 kg/m^2^ versus 22.7 ± 0.1 kg/m^2^). This finding seems to agree with past work in which differences in glucose metabolism between South Asian and European individuals residing near San Francisco (CA, USA) or southeast England were reported [18,27].

### Saturated fatty acids (SFA)

Of the SFA examined, 14:0 (myristic acid) was positively associated with fasting insulin and HOMA-IR in the Caucasian population, and with fasting insulin in the East Asian population. Furthermore, these associations remained significant when examining men and women separately within each ethnicity. This agrees with past work by Lovejoy *et al*., which examined the associations between relative FA levels in the cholesterol ester (CE) and phospholipid (PL) fractions of red blood cells (RBC) and markers of IR (i.e. fasting insulin and glucose, and HOMA-IR) in 38 young men and women [5]. Lovejoy and colleagues reported that of the FA examined, relative abundance of CE 14:0 demonstrated the greatest number of positive associations with markers of IR and that these associations were similarly reflected in the PL fraction [5]. Together, our findings and those of Lovejoy *et al*. suggest that, across multiple ethnicities and within both genders, 14:0 appears to be strongly correlated with markers of IR.

We also found that 18:0 (stearic acid) was positively associated with fasting glucose and HOMA-IR in men and women in our Caucasian population. Similar results were reported by Ebbesson *et al*. who demonstrated that the relative abundance of 18:0 in RBC was positively associated with fasting glucose [6]. Additionally, Wang *et al*. found that 18:0 in CE and PL fractions were positively associated with the development of diabetes in middle-aged adults [4]. Although we observed associations with 18:0 in the Caucasian population, we did not detect associations in East Asian or South Asian subjects. Again, this emphasizes the importance of examining associations between FA and markers of IR in an ethnic-specific fashion.

### Monounsaturated fatty acids (MUFA)

Previous work by Mozaffarian *et al*. demonstrated that the relative abundance (expressed as % FA) of 16:1n9 (palmitoleic acid) in the PL fraction of plasma was positively associated with markers of IR in men (i.e. fasting insulin and glucose, and HOMA-IR), but not women, in a European-majority multi-ethnic population [20]. We could not confirm this trend between 16:1n9 and markers of IR in our population; however, this may be explained by differences in study design. Specifically, we examined total plasma FA whereas this previous study examined FA in plasma PL. Despite this, analyzing total plasma FA has uncovered additional associations with longer-chain MUFA that will ultimately contribute to our understanding of how MUFA may influence markers of IR.

We observed a positive association between 18:1c9 (oleic acid) and fasting glucose in both Caucasian and East Asian populations; however, this association appeared to be stronger in Caucasians. Interestingly, previous studies examining the relationship between 18:1c9 and IR have generated inconsistent results. For example, Ryan *et al*. demonstrated that diets rich in 18:1c9 can reduce IR in T2D adult males [32], while Mayer-Davis *et al*. revealed a positive association between dietary 18:1c9 and IR [33]. Even further uncertainty was provided by Lovejoy *et al*. and Hekmatdoost *et al*., who both reported no evidence of a relationship between 18:1c9 and markers of IR [34,35]. A novel aspect of our study was that we analyzed the relationships between 18:1c9 and markers of IR in a sex-specific manner, which may provide a partial explanation of the discrepancy existing in the literature. We found that the relationship between 18:1c9 and fasting glucose was driven by Caucasian men, not women. We do not believe that this association has been recognized before and underlines potential sex-specific differences regarding the relationship between 18:1c9 and markers of IR.

Interestingly, we observed associations between 18:1t6-8 (sum of 18:1t6, t7, t8) and HOMA-IR in the Caucasian population, which to our knowledge has not been previously reported. Furthermore, this association appeared to be stronger in men compared to women. Although we adjusted our regression models to account for total fat intake, we acknowledge that FFQ can introduce inaccuracies in self-reporting. Therefore, we cannot exclude that this sex difference in 18:1t6-8 may be related to differences in dietary habits (e.g. ruminant milk and meat consumption [36]) that were inaccurately reported by participants.

We also found that 18:1t11 (trans-vaccenic acid) was positively associated with HOMA-IR in Caucasians, but not in East or South Asian populations. This finding is in agreement with a previous study reporting that vaccenic acid levels isolated from serum triglycerides were positively associated with fasting insulin and HOMA-IR in Caucasian males classified as either normoinsulinaemic/normoglycaemic or hyperinsulinaemic/hyperglycaemic [7]. Conversely, Takeuchi *et al*. recently reported that healthy young Japanese men and women (22.8 ± 3.0 yrs) supplemented with 0.6% trans-FA for 4-weeks demonstrated no effect on fasting measures of insulin and glucose [37]. While the findings of these two previous studies may initially appear to conflict, the results of our current research may shed some light on this issue. Indeed, our data suggests that trans-FA are more strongly associated with adverse effects on glucose metabolism in Caucasians, and less so in East and South Asian populations.

### Polyunsaturated fatty acids (PUFA)

In the PUFA class, we found that 18:2n6 (linoleic acid) was positively associated with fasting glucose in our Caucasian population, but not in the East Asian or South Asian groups. Previous studies investigating the relationships between 18:2n6 and markers of IR have often generated inconsistent results. For example, several previous studies have found no evidence of an association between 18:2n6 and markers of IR in diabetic Japanese adults [14] or overweight French adolescents [38]. In contrast, Salomaa *et al*. observed that 18:2n6 in serum CE was lower in diabetic patients compared to healthy controls [39]. Our data therefore adds to the current literature as we have uncovered a novel association with 18:2n6 in a healthy young population; however, it is clear that further investigation of the relationship between 18:2n6 and markers of IR is required.

We also found that 18:2c9t11 (cis-9, trans-11 conjugated linoleic acid) was positively associated with HOMA-IR in Caucasians. These results appear to conflict with previous findings demonstrating a beneficial effect of 18:2c9t11 on insulin sensitivity in middle-aged healthy adults [40]; however, the different ages between these two populations makes it difficult to directly compare the studies. The majority of research examining CLA has been conducted in rodents and has either not focused on specific CLA isoforms or has generated inconsistent results [41,42]. Since findings from rodent models cannot always be extrapolated to humans, this reinforces the need for further examination of the effects of 18:2c9t11 on IR in humans. To our knowledge, this is the first study to demonstrate that the 18:2c9t11 isoform in plasma is positively associated with markers of IR in Caucasians, but that this association does not exist in East Asian and South Asian populations. Closer investigation revealed that 18:2c9t11 was positively associated with HOMA-IR in both Caucasian men and women. However, this relationship was more significant in Caucasian men compared to women, which further points towards a role of sex-specific characteristics on associations between FA and markers of IR.

### Challenges and limitations

We acknowledge that there are several limitations to consider with the present study. Firstly, we did not separate total FA into individual lipid classes, which would provide further insight into dietary habits (i.e. namely FA consumption) in relation to ethnicity and sex. Thus, we were unable to determine whether a FA in a specific lipid fraction was driving the associations with markers of IR. Future investigations examining FA and markers of IR should consider separating total FA into distinct lipid classes, as well as considering different biological materials (e.g. serum or erythrocytes) in order to better understand the relationships between FA and IR. Secondly, the smaller sample size for the South Asian population may have limited our ability to detect potential associations in this ethnic group, and further studies in larger populations are still required. Thirdly, it is important to note that the current study was performed in young healthy adults. As such, it remains to be seen whether these relationships also exist in at-risk (e.g. obese and T2D) populations. Finally, our cross-sectional analysis did not allow us to draw conclusions on cause and effect. To address this, prospective studies are required to examine whether the plasma FA profile in young adults can predict risk for developing T2D later in life.

## Conclusion

In summary, we have demonstrated that circulating levels of specific FA appear to be positively associated with markers of IR. Moreover, we have revealed potential ethnic-differences regarding these relationships (e.g. significant associations were detected in Caucasian and East Asian adults, but not South Asians). Our data also suggests that the associations identified may be stronger in men compared to women. This suggests that underlying sex-specific differences (e.g. hormone levels, adiposity, lifestyle habits, and quantity of muscle mass) may be influencing these associations. Taken together, our results illustrate the significance of investigating associations between FA and IR in an ethnic-specific and sex-specific manner in order to fully understand whether specific plasma FA could contribute to the development of IR and T2D.

## Abbreviations

ALA: α-linolenic acid; BMI: Body mass index; CE: Cholesterol ester; CLA: Conjugated linoleic acid; DHA: Docosahexaenoic acid; EPA: Eicosapentaenoic acid; FA: Fatty acid; FFQ: Food frequency questionnaire; HOMA-IR: Homeostatic model assessment of insulin resistance; IR: Insulin resistance; LA: Linoleic acid; MUFA: Monounsaturated fatty acid; PA: Physical activity; PL: Phospholipid; PUFA: Polyunsaturated fatty acid; RBC: Red blood cells; SFA: Saturated fatty acid; T2D: Type 2 diabetes; TNH: Toronto nutrigenomics and health (study).

## Competing interests

The authors declare that they have no competing interests.

## Authors’ contributions

JCR, MAZ, and DMM designed the study, conducted the analyses, and wrote the manuscript. DEN and AES established the TNH study cohort and collected DNA samples from all participants. SC and DWLM carried out plasma fatty acid analyses. All authors read and approved the final manuscript.
